# Uniformly convergent computational method for singularly perturbed unsteady burger-huxley equation

**DOI:** 10.1016/j.mex.2022.101886

**Published:** 2022-10-22

**Authors:** Imiru Takele Daba, Gemechis File Duressa

**Affiliations:** a Department of Mathematics, Dilla University, Dilla, Ethiopia; b Department of Mathematics, Jimma University, Jimma, Ethiopia

**Keywords:** Quasilinearization technique, Implicit Euler method, Boundary layer, Exponential fitted operator method

## Abstract

This paper deals with the numerical treatment of a singularly perturbed unsteady non-linear Burger-Huxley problem. Due to the simultaneous presence of a singular perturbation parameter and non-linearity in the problem applying classical numerical methods to solve this problem on a uniform mesh are unable to provide oscillation-free results unless they are applied with very fine meshes inside the region. Thus, to resolve this issue, a uniformly convergent computational scheme is proposed. The scheme is formulated:

• First, the non-linear singularly perturbed problem is linearized using the Newton-Raphson-Kantorovich quasilinearization technique.
• The resulting linear singularly perturbed problem is semi-discretized in time using the implicit Euler method to yield a system of singularly perturbed ordinary differential equations in space.
• Finally, the system of singularly perturbed ordinary differential equations are solved using fitted exponential cubic spline method.

The stability and uniform convergence of the proposed scheme are investigated. The scheme is stable and ε−uniformly convergent with first order in time and second order in space directions. To validate the applicability of the proposed scheme several test examples are considered. The obtained numerical results depict that the proposed scheme provides more accurate results than some methods available in the literature.

## Graphical abstract

**Figure:**
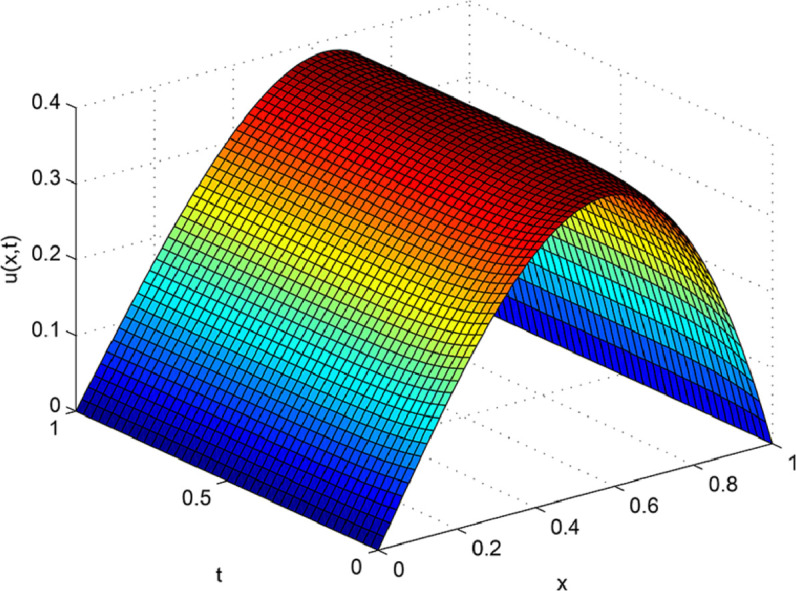

**Specifications table**

**Table:** 

Subject area	Mathematics and Statistics
More specific subject area	Numerical Analysis
Method Name	Uniformly Convergent Computational Method for Singularly Perturbed Unsteady Burger-Huxley Equation
Name and reference of	L.B. Liu, Y. Liang, J. Zhang, X. Bao. A robust
original method	adaptive grid method for singularly perturbed
	burger-huxley equations. Electronic Research Archive
	176 28 (4) (2020) 1439. doi: 10.3934/era.2020076
Resource availability	MATLAB R2013a software package

## Introduction

Consider one-dimensional unsteady singularly perturbed Burger-Huxley equation on the domain $\Upsilon =\chi_{z}\times \chi_{t}=\left(0,1\right)\times \left(0,T\right]$ of the form:

(1.1) $$\left\{ \begin{array}{c} {£}_{\varepsilon }ℑ\left(z,t\right)=\frac{\partial ℑ}{\partial t}-\varepsilon \frac{\partial^{2}ℑ}{\partial z^{2}}+\alpha ℑ\frac{\partial ℑ}{\partial z}-\theta \left(1-ℑ\right)\left(ℑ-\lambda \right)=0,\left(z,t\right)\in \Upsilon ,\\ ℑ\left(z,0\right)={ℑ}_{0}\left(z\right),z\in {\bar{\chi }}_{z},\\ ℑ\left(0,t\right)=\Theta_{0}\left(t\right),ℑ\left(1,t\right)=\Theta_{1}\left(t\right),t\in \left(0,T\right], \end{array}\right.$$

where ε is a small singular perturbation parameter such that 0<ε≪1 and α≥1,θ≥0,λ∈(0,1). The functions $\Theta_{0}\left(t\right),\Theta_{1}\left(t\right)$ and ${ℑ}_{0}\left(z\right)$ are assumed to be sufficiently smooth, bounded and independent of ε. The above Eq.  (1.1) shows a prototype model for describing the interaction between non-linear convection effects, reaction mechanisms, and diffusion transport. This equation has many intriguing phenomena such as bursting oscillation [5], population genetics [1], interspike [24], bifurcation and chaos [34]. Several membrane models based on the dynamics of potassium and sodium ion fluxes found in [22].

Many researchers have made efforts to construct analytical and numerical methods for Burger equations, for instance, [6], [8], [9], [10], [11], [16], [17], [19], [20], [21], [25], [28], [29], [32] and references therein. A Burger-Huxley equation in which the highest order derivative is multiplied by a small perturbation parameter ε (0<ε<<1) is termed as the singularly perturbed Burger-Huxley equation. Due to the presence of perturbation parameter ε the solution reveals boundary/ sharp interior layer(s), and it is not easy to find a stable numerical approximation.

The methods suggested by [6], [8], [9], [10], [11], [16], [17], [19], [20], [21], [25], [28], [29], [32] and other classical numerical methods on a uniform mesh fail to approximate the singularly perturbed Burger-Huxley equation. They require an unacceptably large number of mesh points to sustain the approximation because the mesh width depends on the ε. This limitation of the conventional numerical methods has encouraged researchers to develop robust numerical techniques that perform well enough independent of the ε. [14] presented a uniformly convergent finite difference method (FDM) for the problem (1.1). [7] developed a numerical method that comprises of an implicit-Euler method to discretize in temporal direction on a uniform mesh and a monotone hybrid finite difference operator to discretize the spatial variable with a piecewise uniform Shishkin mesh.

A robust numerical method that consists of backward-Euler scheme on a uniform mesh to approximate time derivative and upwind FDM on an adaptive nonuniform grid is used for space derivative of Eq.  (1.1) is suggested by [23]. [12], [13] considered a similar problem as in [23] and proposed a parameter uniform numerical method based on fitted operator techniques. Very recently, [3] developed fitted operator finite difference method for singularly perturbed Burgers’ initial-boundary value problem.

Nevertheless, the numerical treatment of problem (1.1) has seen little development. The purpose of this work is to formulate and analyze an ε−uniformly convergent numerical method for solving the problem (1.1). The novelty of the presented method, unlike Shishkin mesh developed by [30] and Bakhvalov mesh developed by [2], does not require a priori information about the location and width of the boundary layer.

The methods developed by [7], [12], [13], [14], [23] for Eq.  (1.1) are based on finite difference method. In comparison with the finite difference methods, spline solution has its own advantages. For instance, once the solution has been computed, the information required for spline interpolation between mesh points is available [15]. Also, splines are a simpler and more practical way to solve boundary-value problems than finite difference methods [18]. This is particularly important when the solution of the boundary-value problem is required at various locations in the interval [a,b]. This provides the motivation for our work on using fitted exponential spline method for solving the problem under consideration.

## A priori estimates for the solution of the continuous problem

Lemma 2.1 **Continuous Maximum principle**

*Let* $ℑ\in C^{2,1}\left(\bar{\Upsilon }\right)$ *, be a smooth function such that* ℑ(z,t)≥0,∀(z,t)∈∂Υ *and* ${£}_{\varepsilon }ℑ\left(z,t\right)\geq 0,\forall \left(z,t\right)\in \Upsilon$ *. Then* $ℑ\left(z,t\right)\geq 0,\forall \left(z,t\right)\in \bar{\Upsilon }$ *.*

Proof See [7]. □

An immediate consequence of Lemma 2.1 for the solution of problem (1.1) gives the following uniform stability estimate.

Lemma 2.2 **Continuous stability estimate**

*Let*ℑ(z,t)*be the solution of problem*(1.1)*, then we have,*

$${∥ℑ∥}_{\bar{\Upsilon }}\leq T{∥{ℑ}_{0}∥}_{\Lambda_{i}}+{∥ℑ∥}_{\partial \Upsilon },$$

*where*$\Lambda_{i}\left\{\left(z,t\right):t=0,z\in \left[0,1\right]\right\}$*.*

Proof See [7]. □

## Formulation of the numerical scheme

### Quasi-linearization

Let’s rewrite Eq.  (1.1) as

(3.1) $$\left\{ \begin{array}{c} {£}_{\varepsilon }ℑ\left(z,t\right)=\left(\frac{\partial ℑ}{\partial t}-\varepsilon \frac{\partial^{2}ℑ}{\partial z^{2}}\right)\left(z,t\right)=\zeta \left(z,t,ℑ\left(z,t\right),{ℑ}_{z}\left(z,t\right)\right),\left(z,t\right)\in \Upsilon ,\\ ℑ\left(z,0\right)={ℑ}_{0}\left(z\right),z\in {\bar{\chi }}_{z},\\ ℑ\left(0,t\right)=\Theta_{0}\left(t\right),ℑ\left(1,t\right)=\Theta_{1}\left(t\right),t\in \left(0,T\right], \end{array}\right.$$

where $\zeta \left(z,t,ℑ\left(z,t\right),{ℑ}_{z}\left(z,t\right)\right)=-\alpha ℑ\frac{\partial ℑ}{\partial z}+\theta \left(1-ℑ\right)\left(ℑ-\lambda \right)$ is the non-linear function of $z,t,ℑ\left(z,t\right),{ℑ}_{z}\left(z,t\right)$.

To linearize the semi-linear term of Eq.  (1.1), we choose the reasonable initial approximation for the function ℑ(z,t) in the term $\zeta (z,t,ℑ\left(z,t\right),{ℑ}_{z}\left(z,t\right))$, and denote it as ${ℑ}^{0}\left(z,t\right)$ that satisfy both initial and boundary conditions which is obtained by separation of variables method of the homogeneous part of the problem under consideration and is given by [13]:

(3.2) $${ℑ}^{0}\left(z,t\right)={ℑ}_{0}\left(z\right)\exp\left(-\pi^{2}t\right).$$

Thus, the nonlinear term $\zeta (z,t,ℑ\left(z,t\right),{ℑ}_{z}\left(z,t\right))$ of Eq. (3.1) can be linearized by applying the Newton-Raphson- Kantorovich approximation approach as

(3.3) $$\begin{array}{ccc} & & \zeta \left(z,t,{ℑ}^{\left(k+1\right)}\left(z,t\right),{ℑ}_{z}^{\left(k+1\right)}\left(z,t\right)\right)≅\zeta \left(z,t,{ℑ}^{\left(k\right)}\left(z,t\right),{ℑ}_{z}^{\left(k\right)}\left(z,t\right)\right)\\ & & +\;\left({ℑ}^{\left(k+1\right)}\left(z,t\right)-{ℑ}^{\left(k\right)}\left(z,t\right)\right)\zeta_{ℑ}{|}_{(}ℑ,t,{ℑ}^{\left(k\right)}\left(z,t\right),{ℑ}_{z}^{\left(k\right)}\left(z,t\right))\\ & & +\;\left({ℑ}_{z}^{\left(k+1\right)}\left(z,t\right)-{ℑ}_{z}^{\left(k\right)}\left(ℑ,t\right)\right)\zeta_{{ℑ}_{z}}{|}_{(}z,t,{ℑ}^{\left(k\right)}\left(z,t\right),{ℑ}_{z}^{\left(k\right)}\left(z,t\right))+\cdots . \end{array}$$

Substitution of Eq.  (3.3) into Eq. (3.1) and after simplification the following linear second-order differential equations obtained:

(3.4) $$\left\{ \begin{array}{c} {£}_{\varepsilon }{ℑ}^{\left(k+1\right)}\left(z,t\right)={ℑ}_{t}^{\left(k+1\right)}\left(z,t\right)-\varepsilon {ℑ}_{zz}^{\left(k+1\right)}\left(z,t\right)+\varpi \left(z,t\right){ℑ}_{z}^{\left(k+1\right)}\left(z,t\right)+q\left(z,t\right){ℑ}^{\left(k+1\right)}\left(z,t\right)\\ \;\;\;\;\;\;\;\;\;\;\;\;\;\;\;\;\;\;\;=v(z,t),\\ {ℑ}^{\left(k+1\right)}\left(z,0\right)={ℑ}_{0}\left(z\right),z\in {\bar{\chi }}_{z},\\ {ℑ}^{\left(k+1\right)}\left(0,t\right)=\Theta_{0}\left(t\right),t\in {\bar{\chi }}_{t},\\ {ℑ}^{\left(k+1\right)}\left(1,t\right)=\Theta_{1}\left(t\right),t\in {\bar{\chi }}_{t}, \end{array}\right.$$

where

$$\begin{array}{ccc} \varpi (z,t) & = & -\zeta_{{ℑ}_{z}}{|}_{(}z,t,{ℑ}^{\left(k\right)}\left(z,t\right),{ℑ}_{z}^{\left(k\right)}\left(z,t\right)),\\ q(z,t) & = & -\zeta_{ℑ}{|}_{(}z,t,{ℑ}^{\left(k\right)}\left(z,t\right),{ℑ}_{z}^{\left(k\right)}\left(z,t\right)),\\ v(z,t) & = & \zeta \left(z,t,{ℑ}^{\left(k\right)}\left(z,t\right),{ℑ}_{z}^{\left(k\right)}\left(z,t\right)\right)-{ℑ}^{\left(k\right)}\zeta_{ℑ}{|}_{(}z,t,{ℑ}^{\left(k\right)}\left(z,t\right),{ℑ}_{z}^{\left(k\right)}\left(z,t\right))\\ & & -\;{ℑ}_{z}^{\left(k\right)}\zeta_{{ℑ}_{z}}{|}_{(}z,t,{ℑ}^{\left(k\right)}\left(z,t\right),{ℑ}_{z}^{\left(k\right)}\left(z,t\right)). \end{array}$$

### Temporal semi-discretization

To discretize the time variable for Eq.  (3.4), we use the implicit Euler method with uniform time step $\tau ,\;\Upsilon_{\tau }^{M}=\left\{t_{j}=j\tau ,\forall j=0,2,3,\cdots ,M,\tau =1/M\right\}$, which is given by

(3.5) $$\left\{ \begin{array}{c} \left(1+\tau {£}_{\varepsilon }^{M}\right){\hat{ℑ}}^{k+1}\left(z,t_{j+1}\right)=-\varepsilon {\hat{ℑ}}_{zz}^{k+1}\left(z,t_{j+1}\right)+\varpi \left(z,t_{j+1}\right){\hat{ℑ}}_{z}^{k+1}\left(z,t_{j+1}\right)+\\ \;\;\;\;\;\;\;\;\;\;\;\;\;\;\;\;\;\;\;\;\;\;\;\;\;\;\;\;\;q\left(z,t_{j+1}\right){\hat{ℑ}}^{k+1}\left(z,t_{j+1}\right)-v\left(z,t_{j+1}\right)={\hat{ℑ}}^{k+1}\left(z,t_{j}\right),\\ {\hat{ℑ}}^{k+1}\left(z,0\right)={\hat{ℑ}}_{0}^{k+1}\left(z\right),z\in {\bar{\chi }}_{z},\\ {\hat{ℑ}}^{k+1}\left(0,t_{j+1}\right)=\Theta_{0}\left(t_{j+1}\right),0\leq j\leq M-1,\\ {\hat{ℑ}}^{k+1}\left(1,t_{j+1}\right)=\Theta_{1}\left(t_{j+1}\right),0\leq j\leq M-1. \end{array}\right.$$

Since, $\varpi \left(z,t_{j+1}\right)\geq \varpi^{*}>0,\text{and}\;q\left(z,t_{j+1}\right)\geq q^{*}>0,z\in {\bar{\chi }}_{z}$ Eq.  (3.5) exhibits boundary located at z=1 and admits a unique solution. Clearly, the operator $\left(1+\tau {£}_{\varepsilon }^{M}\right)$ satisfies the maximum principle, which ensures the stability of the semi-discrete (3.5).

Lemma 3.1 **Local Error Estimate**

*If*$\left|\frac{\partial^{n}ℑ\left(z,t\right)}{\partial t^{n}}\right|\leq C,\;\forall \left(z,t\right)\in \bar{\Upsilon },n=0,1,2$*, then the local error estimate in the temporal direction satisfies*

$${∥e_{j+1}∥}_{\infty }\leq C\;\tau^{2},$$

where C is a positive constant independent of ε and Δt.

Proof The use of Taylor’s series expansion for ${\hat{ℑ}}^{k+1}\left(z,t_{j+1}\right)$ provides

$${\hat{ℑ}}^{k+1}\left(z,t_{j+1}\right)={\hat{ℑ}}^{k+1}\left(z,t_{j}\right)+\tau {\hat{ℑ}}_{t}^{k+1}\left(z,t_{j}\right)+O\left({\left(\tau \right)}^{2}\right).$$

This implies

(3.6) $$\frac{{\hat{ℑ}}^{k+1}\left(z,t_{j+1}\right)-{\hat{ℑ}}^{k+1}\left(z,t_{j}\right)}{\tau }={\hat{ℑ}}_{t}^{k+1}\left(z,t_{j}\right)+O\left(\tau \right).$$

Substituting Eq.  (3.4) into Eq.  (3.6) yields

(3.7) $$\begin{array}{ccc} & & \frac{{\hat{ℑ}}^{k+1}\left(z,t_{j+1}\right)-{\hat{ℑ}}^{k+1}\left(z,t_{j}\right)}{\tau }=-\left(-\varepsilon {\hat{ℑ}}_{zz}^{k+1}\left(z,t_{j}\right)+\varpi \left(z,t_{j}\right){\hat{ℑ}}_{z}^{k+1}\left(z,t_{j}\right)\right)\\ & & -\;\left(q\left(z,t_{j}\right){\hat{ℑ}}^{k+1}\left(z,t_{j}\right)-v\left(z,t_{j}\right)\right)+O\left(\tau \right)\\ & & \Rightarrow \left(1+\tau {£}_{\varepsilon }^{M}\right){\hat{ℑ}}^{k+1}\left(z,t_{j+1}\right)-\tau v\left(z,t_{j}\right)+{\hat{ℑ}}^{k+1}\left(z,t_{j}\right)=O{\left(\tau \right)}^{2}). \end{array}$$

Subtracting Eq.  (3.5) from Eq.  (3.7), and the local truncation error $e_{j+1}={\hat{ℑ}}^{k+1}\left(z,t_{j+1}\right)-{\hat{ℑ}}^{k+1}\left(z,t_{j+1}\right)$ at (j+1)th is the solution of a boundary problem

(3.8) $$\left(1+\tau {£}_{\varepsilon }^{M}\right)e_{j+1}=O\left({\left(\tau \right)}^{2}\right),e_{j+1}\left(0\right)=0=e_{j+1}\left(1\right),$$

where ${\hat{ℑ}}^{k+1}\left(z,t_{j+1}\right)$ is the solution of the boundary value problem (3.5).

Hence, applying the maximum principle on the operator gives

$${∥e_{j+1}∥}_{\infty }\leq C\;\tau^{2}.$$

 □

Lemma 3.2 **Global Error Estimate**

*Under the hypothesis of Lemma*3.1*, the global error estimate in the temporal direction is given by*

$${∥E_{j}∥}_{\infty }\leq C\;\tau ,\;\forall \;j\leq T/\tau .$$

Proof The use of Lemma 3.1 yields

$$\begin{array}{ccc} {∥E_{j}∥}_{\infty } & = & {∥\sum_{i=1}^{j}e_{i}∥}_{\infty },\;\;\;j\leq T/\tau \\ & \leq & {∥e_{1}∥}_{\infty }+{∥e_{2}∥}_{\infty }+{∥e_{3}∥}_{\infty }+\cdots +{∥e_{j}∥}_{\infty }\\ & \leq & c_{1}j\;\tau^{2}\left(\text{by}\;\;\;\text{Lemma}\;3.1\right)\\ & \leq & c_{1}j\tau \tau \\ & \leq & c_{1}T\tau \left(j\tau \leq T\right)\\ & \leq & C\;\tau , \end{array}$$

where $c_{1},C$ are positive constants independent of ε and τ. □

Lemma 3.3 *The bound on the derivative of the solution*${\hat{ℑ}}^{k+1}\left(z\right)$*of the* Eq. (3.5) *is given by*

$${∥\frac{\partial^{m}{\hat{ℑ}}^{k+1}\left(z,t_{j+1}\right)}{\partial z^{m}}∥}_{{\bar{\chi }}_{z}}\leq \left(C\right)\left(1+\varepsilon^{-m}\exp\left(\frac{-(\varpi^{*}\left(1-z\right))}{\varepsilon }\right)\right),0\leq m\leq 4.$$

Proof See [7]. □

### Spatial Semi-discretization

The space domain [0,1] is divided into N equidistant mesh with the spatial step size ℓ such that

$$\Upsilon_{\ell }^{N}=\left\{z_{i}=i\ell ,\forall i=1,2,3,\cdots ,N,z_{0}=0,z_{N}=1,\ell =1/N\right\}.$$

Let us rewrite Eq.  (3.5) for i=0,1,⋯,N, and j=0,1⋯,M−1 as

(3.9) $$\begin{array}{ccc} & & -\varepsilon {\hat{ℑ}}_{zz}^{k+1}\left(z_{i},t_{j+1}\right)+\varpi \left(z_{i},t_{j+1}\right){\hat{ℑ}}_{z}^{k+1}\left(z_{i},t_{j+1}\right)+r\left(z_{i},t_{j+1}\right){\hat{ℑ}}^{k+1}\left(z_{i},t_{j+1}\right)=\varphi \left(z_{i},t_{j+1}\right),\\ & & \hat{ℑ}\left(0,t_{j+1}\right)=\Theta_{0}\left(t_{j+1}\right),\hat{ℑ}\left(1,t_{j+1}\right)=\Theta_{1}\left(t_{j+1}\right), \end{array}$$

where $r\left(z_{i},t_{j+1}\right)=\left(q\left(z_{i},t_{j+1}\right)+\frac{1}{\tau }\right)$ and $\varphi \left(z_{i},t_{j+1}\right)=v\left(z_{i},t_{j+1}\right)+\frac{{\hat{ℑ}}^{k+1}\left(z_{i},t_{j}\right)}{\tau }$.

To solve Eq.  (3.9), we use an exponential cubic spline method as discussed below. Let ${\hat{ℑ}}_{i}^{j+1}$ be an approximation to the exact solution ${\hat{ℑ}}^{k+1}\left(z_{i},t_{j+1}\right)$ of Eq.  (3.9) obtained by the segment $S_{▵}\left(z\right)$ passing through the points $\left(z_{i},{\hat{ℑ}}_{i}^{j+1}\right)$ and $\left(z_{i+1},{\hat{ℑ}}_{i+1}^{j+1}\right)$. Each mixed spline segment $S_{▵}^{j+1}\left(z\right)$ has the form [33]:

(3.10) $$S_{▵}^{j+1}\left(z\right)=A_{i}e^{\psi (z-z_{i})}+B_{i}e^{-\psi (z-z_{i})}+G_{i}\left(z-z_{i}\right)+D_{i},i=0,1,2,\cdots ,N,$$

where, $A_{i},B_{i},G_{i},$ and $D_{i}$ are constants and ψ≠0 is a free parameter that will be used to enhance the accuracy of the method. To find the unknown coefficients in Eq.  (3.10), let us denote:

(3.11) $$\begin{array}{c} S_{▵}^{j+1}\left(z_{i}\right)={\hat{ℑ}}_{i}^{j+1},\;\;\;\;\;\;\;\;S_{▵}^{j+1}\left(z_{i+1}\right)={\hat{ℑ}}_{i+1}^{j+1},\\ \frac{d^{2}S_{▵}^{j+1}\left(z_{i}\right)}{dz^{2}}=\Xi_{i},\;\;\;\;\;\;\;\;\;\;\;\frac{d^{2}S_{▵}^{j+1}\left(z_{i+1}\right)}{dz^{2}}=\Xi_{i+1}. \end{array}$$

Differentiating twice both sides of Eq.  (3.10) with respect to z yields

(3.12) $$\frac{d^{2}S_{▵}^{j+1}\left(z\right)}{dz^{2}}=\psi^{2}\left(A_{i}e^{\psi (z-z_{i})}+B_{i}e^{-\psi (z-z_{i})}\right).$$

Using the relation in Eq.  (3.11) into (3.12) at $z=z_{i}$,

$$\frac{d^{2}S_{▵}^{j+1}\left(z_{i}\right)}{dz^{2}}=\Xi_{i}=\psi^{2}\left(A_{i}+B_{i}\right)$$

and it yields

(3.13) $$A_{i}=\frac{\ell^{2}\Xi_{i}}{\mu^{2}}-B_{i},$$

where μ=ψℓ and i=0,1,2,⋯,N.

Again substituting Eq.  (3.11) into (3.12) at $z=z_{i+1}$,

$$\frac{d^{2}S_{▵}^{j+1}\left(z_{i+1}\right)}{dz^{2}}=\Xi_{i+1}=\psi^{2}\left(A_{i}e^{\mu }+B_{i}e^{-\mu }\right)$$

and it gives

(3.14) $$A_{i}=e^{-\mu }\left(\frac{\ell^{2}\Xi_{i+1}}{\mu^{2}}\right)-B_{i}e^{-2\mu }.$$

From Eqs. (3.13) and (3.14),

(3.15) $$A_{i}=\frac{\ell^{2}\left(\Xi_{i+1}-\Xi_{i}e^{-\mu }\right)}{2\mu^{2}\sinh\left(\mu \right)}\;\;\text{and}\;\;\;B_{i}=\frac{\ell^{2}\left(\Xi_{i}e^{\mu }-\Xi_{i+1}\right)}{2\mu^{2}\sinh\left(\mu \right)}.$$

Taking Eq.  (3.11) into Eq.  (3.10) at $z=z_{i}$ and $z=z_{i+1}$,

$$S_{▵}^{j+1}\left(z_{i}\right)={\hat{ℑ}}_{i}^{j+1}=A_{i}+B_{i}+D_{i},\;\text{and}\;S_{▵}^{j+1}\left(z_{i+1}\right)={\hat{ℑ}}_{i+1}^{j+1}=A_{i}e^{\mu }+B_{i}e^{-\mu }+G_{i}\ell +D_{i},$$

and which implies

(3.16) $$G_{i}=\frac{\left({\hat{ℑ}}_{i+1}^{j+1}-{\hat{ℑ}}_{i}^{j+1}\right)}{\ell }-\frac{\ell \left(\Xi_{i+1}-\Xi_{i}\right)}{\mu^{2}},\;\;\;\text{and}\;\;\;D_{i}={\hat{ℑ}}_{i}^{j+1}-\left(\frac{\ell^{2}}{\mu^{2}}\Xi_{i}\right).$$

Using the continuity condition of the first derivative at $z=z_{i}$, that is $S_{▵-1}\left(z_{i}\right)$ $=S_{▵}\left(z_{i}\right)$ yields

(3.17) $$\ell^{2}\left(\omega_{1}\Xi_{i-1}+2\omega_{2}\Xi_{i}+\omega_{1}\Xi_{i+1}\right)=\left({\hat{ℑ}}_{i-1}^{j+1}-2{\hat{ℑ}}_{i}^{j+1}+{\hat{ℑ}}_{i+1}^{j+1}\right),i=1,2,\cdots ,N-1,$$

where

$$\omega_{1}=\left(\frac{\sinh(\mu )-\mu }{\mu^{2}\sinh\left(\mu \right)}\right)\;\;\text{and}\;\;\omega_{2}=\left(\frac{2\mu \cosh\left(\mu \right)-2\sinh\left(\mu \right)}{\mu^{2}\sinh\left(\mu \right)}\right).$$

Equation  (3.9) can be rewritten as

(3.18) $$\left\{ \begin{array}{c} \varepsilon \Xi_{i-1}=\varpi^{j+1}\left(z_{i-1}\right){\hat{ℑ}}_{z}^{j+1}\left(z_{i-1}\right)+r^{j+1}\left(z_{i-1}\right){\hat{ℑ}}^{j+1}\left(z_{i-1}\right)-\varphi^{j+1}\left(z_{i-1}\right),\\ \varepsilon \Xi_{i}=\varpi^{j+1}\left(z_{i}\right){\hat{ℑ}}_{z}^{j+1}\left(z_{i}\right)+r^{j+1}\left(z_{i}\right){\hat{ℑ}}^{j+1}\left(z_{i}\right)-\varphi^{j+1}\left(z_{i}\right),\\ \varepsilon \Xi_{i+1}=\varpi^{j+1}\left(z_{i+1}\right){\hat{ℑ}}_{z}^{j+1}\left(z_{i+1}\right)+r^{j+1}\left(z_{i+1}\right){\hat{ℑ}}^{j+1}\left(z_{i+1}\right)-\varphi^{j+1}\left(z_{i+1}\right), \end{array}\right.$$

where we approximate ${\hat{ℑ}}_{z}^{j+1}\left(z_{i-1}\right),{\hat{ℑ}}_{z}^{j+1}\left(z_{i}\right)$ and ${\hat{ℑ}}_{z}^{j+1}\left(z_{i+1}\right)$ as

(3.19) $$\left\{ \begin{array}{c} {\hat{ℑ}}_{z}^{j+1}\left(z_{i-1}\right)≅\frac{-{\hat{ℑ}}_{i+1}^{j+1}+4{\hat{ℑ}}_{i}^{j+1}-3{\hat{ℑ}}_{i-1}^{j+1}}{2\ell }.\\ {\hat{ℑ}}_{z}^{j+1}\left(z_{i}\right)≅\frac{{\hat{ℑ}}_{i+1}^{j+1}-{\hat{ℑ}}_{i-1}^{j+1}}{2\ell },\\ {\hat{ℑ}}_{z}^{j+1}\left(z_{i+1}\right)≅\frac{3{\hat{ℑ}}_{i+1}^{j+1}-4{\hat{ℑ}}_{i}^{j+1}+{\hat{ℑ}}_{i-1}^{j+1}}{2\ell }. \end{array}\right.$$

Substituting Eq.  (3.19) into (3.18) gives

(3.20) $$\left\{ \begin{array}{c} \varepsilon \Xi_{i-1}=\varpi^{j+1}\left(z_{i-1}\right)\left(\frac{-{\hat{ℑ}}_{i+1}^{j+1}+4{\hat{ℑ}}_{i}^{j+1}-3{\hat{ℑ}}_{i-1}^{j+1}}{2\ell }\right)+r^{j+1}\left(z_{i-1}\right){\hat{ℑ}}^{j+1}\left(z_{i-1}\right)-\varphi^{j+1}\left(z_{i-1}\right),\\ \varepsilon \Xi_{i}=\varpi^{j+1}\left(z_{i}\right)\left(\frac{{\hat{ℑ}}_{i+1}^{j+1}-{\hat{ℑ}}_{i-1}^{j+1}}{2\ell }\right)+r^{j+1}\left(z_{i}\right){\hat{ℑ}}^{j+1}\left(z_{i}\right)-\varphi^{j+1}\left(z_{i}\right),\\ \varepsilon \Xi_{i+1}=\varpi^{j+1}\left(z_{i+1}\right)\left(\frac{3{\hat{ℑ}}_{i+1}^{j+1}-4{\hat{ℑ}}_{i}^{j+1}+{\hat{ℑ}}_{i-1}^{j+1}}{2\ell }\right)+r^{j+1}\left(z_{i+1}\right){\hat{ℑ}}^{j+1}\left(z_{i+1}\right)-\varphi^{j+1}\left(z_{i+1}\right). \end{array}\right.$$

Substituting Eq.  (3.20) into Eq.  (3.17) and rearranging as

(3.21) $$\begin{array}{ccc} & & \varepsilon \left(\frac{{\hat{ℑ}}_{i-1}^{j+1}-2{\hat{ℑ}}_{i}^{j+1}+{\hat{ℑ}}_{i+1}^{j+1}}{\ell^{2}}\right)=\left(-\frac{3\omega_{1}\varpi_{i-1}^{j+1}}{2\ell }-\frac{\omega_{2}\varpi_{i}^{j+1}}{\ell }+\frac{\omega_{1}\varpi_{i+1}^{j+1}}{2\ell }+\omega_{1}r_{i-1}^{j+1}\right){\hat{ℑ}}_{i-1}^{j+1}\\ & & +\;\left(\frac{2\omega_{1}\varpi_{i-1}^{j+1}}{\ell }-\frac{2\omega_{1}\varpi_{i+1}^{j+1}}{\ell }+2\omega_{2}r_{i}^{j+1}\right){\hat{ℑ}}_{i}^{j+1}\\ & & +\;\left(-\frac{\omega_{1}\varpi_{i-1}^{j+1}}{2\ell }+\frac{\omega_{2}\varpi_{i}^{j+1}}{\ell }+\frac{3\omega_{1}\varpi_{i-1}^{j+1}}{2\ell }+\omega_{1}r_{i+1}^{j+1}\right){\hat{ℑ}}_{i+1}^{j+1}=\left(\omega_{1}\varphi_{i-1}^{j+1}+\omega_{2}\varphi_{i}^{j+1}+\omega_{1}\varphi {j+1}_{i+1}\right). \end{array}$$

Now, introducing a fitting factor σ(ρ), which is used to handle the effect of the ε on solution behavior, into the above Eq. (3.21) provides

(3.22) $$\begin{array}{ccc} & & \left(\frac{-\sigma \left(\rho \right)}{\rho }-\frac{3\omega_{1}\varpi_{i-1}^{j+1}}{2}-\omega_{2}\varpi_{i}^{j+1}+\frac{\omega_{1}\varpi_{i+1}^{j+1}}{2}+\ell \omega_{1}r_{i-1}^{j+1}\right){\hat{ℑ}}_{i-1}^{j+1}\\ & & +\;\left(\frac{2\sigma \left(\rho \right)}{\rho }+2\omega_{1}\varpi_{i-1}^{j+1}-2\omega_{1}\varpi_{i+1}^{j+1}+2\ell \omega_{2}r_{i}^{j+1}\right){\hat{ℑ}}_{i}^{j+1}\\ & & +\;\left(\frac{-\sigma \left(\rho \right)}{\rho }-\frac{\omega_{1}\varpi_{i-1}^{j+1}}{2}+\omega_{2}\varpi_{i}^{j+1}+\frac{3\omega_{1}\varpi_{i-1}^{j+1}}{2}+\ell \omega_{1}r_{i+1}^{j+1}\right){\hat{ℑ}}_{i+1}^{j+1}\\ & & =\ell \left(\omega_{1}\varphi_{i-1}^{j+1}+\omega_{2}\varphi_{i}^{j+1}+\omega_{1}\varphi_{i+1}^{j+1}\right), \end{array}$$

where $\rho =\frac{\ell }{\varepsilon }$.

Taking the limit of Eq.  (3.22) as ℓ→0:

(3.23) $$\begin{array}{ccc} & & s\lim_{\ell \to 0}\left(\frac{\sigma \left(\rho \right)}{\rho }\right)\left({\hat{ℑ}}^{j+1}\left(i\ell -\ell \right)-2{\hat{ℑ}}^{j+1}\left(i\ell \right)+{\hat{ℑ}}^{j+1}\left(i\ell +\ell \right)\right)\\ & & +\;\left(\omega_{1}+\omega_{2}\right)\lim_{\ell \to 0}\left(\varpi^{j+1}\left(i\ell \right)\right)\left({\hat{ℑ}}^{j+1}\left(i\ell +\ell \right)-{\hat{ℑ}}^{j+1}\left(i\ell -\ell \right)\right)=0. \end{array}$$

For problems with a layer at the right end of the interval, using the theory of singular perturbations, the solution of Eq.  (3.9) takes the form [27]

(3.24) $${\hat{ℑ}}^{j+1}\left(z\right)\approx {ℑ}_{o}^{j+1}\left(z\right)+\frac{\varpi^{j+1}\left(1\right)}{\varpi^{j+1}\left(z\right)}\left(\Theta_{1}^{j+1}-{\hat{ℑ}}_{0}^{j+1}\left(1\right)\right)\exp\left(-\varpi^{j+1}\left(z\right)\frac{\left(1-z\right)}{\varepsilon }\right)+O\left(\varepsilon \right),$$

where ${ℑ}_{0}^{j+1}\left(z\right)$ is the solution of the reduced problem

$$\varpi^{j+1}\left(z\right)\frac{\partial {\hat{ℑ}}_{0}^{j+1}\left(z\right)}{\partial z}+r^{j+1}\left(z\right){\hat{ℑ}}_{0}^{j+1}\left(z\right)=\varphi^{j+1}\left(z\right),\;\text{with}\;{\hat{ℑ}}_{0}^{j+1}\left(1\right)=\Theta_{1}^{j+1}.$$

Considering Taylor’s series expansion for $\varpi^{j+1}\left(z\right)$ about the point z=1 and restricting to their first terms, Eq.  (3.24) becomes

(3.25) $${\hat{ℑ}}^{j+1}\left(z\right)\approx {\hat{ℑ}}_{0}^{j+1}\left(z\right)+\left(\Theta_{1}^{j+1}-{\hat{ℑ}}_{0}^{j+1}\left(1\right)\right)\exp\left(-\varpi^{j+1}\left(1\right)\frac{\left(1-z\right)}{\varepsilon }\right)+O\left(\varepsilon \right).$$

Equation  (3.25) at $z_{i}=i\ell$ and as ℓ→0 becomes

$$\left\{ \begin{array}{c} \lim_{\ell \to 0}{\hat{ℑ}}^{j+1}\left(i\ell \right)\approx {\hat{ℑ}}_{0}^{j+1}\left(0\right)+\left(\Theta_{1}^{j+1}-{\hat{ℑ}}_{0}^{j+1}\left(1\right)\right)\exp\left(-\varpi^{j+1}\left(1\right)\left(\frac{1}{\varepsilon }-i\rho \right)\right)+O\left(\varepsilon \right),\;\;\;\;\;\;\\ \lim_{\ell \to 0}{\hat{ℑ}}^{j+1}\left(\left(i-1\right)\ell \right)\approx {\hat{ℑ}}_{0}^{j+1}\left(0\right)+\left(\Theta_{1}^{j+1}-{\hat{ℑ}}_{0}^{j+1}\left(1\right)\right)\exp\left(-\varpi^{j+1}\left(1\right)\left(\frac{1}{\varepsilon }-i\rho +\rho \right)\right)+O\left(\varepsilon \right),\\ \lim_{\ell \to 0}{\hat{ℑ}}^{j+1}\left(\left(i+1\right)\ell \right)\approx {\hat{ℑ}}_{0}^{j+1}\left(0\right)+\left(\Theta_{1}^{j+1}-{\hat{ℑ}}_{0}^{j+1}\left(1\right)\right)\exp\left(-\varpi^{j+1}\left(1\right)\left(\frac{1}{\varepsilon }-i\rho -\rho \right)\right)+O\left(\varepsilon \right). \end{array}\right.$$

Plugging the above equations into Eq.  (3.23), we obtain the required fitting factor

(3.26) $$\sigma \left(\rho \right)=\varpi^{j+1}\left(0\right)\rho \left(\omega_{1}+\omega_{2}\right)\coth\left(\frac{\varpi^{j+1}\left(1\right)\rho }{2}\right).$$

Finally, using Eqs. (3.22) and Eq.  (3.26):

(3.27) $${£}_{\varepsilon }^{N,M}{\hat{ℑ}}_{i}^{j+1}=H_{i}^{j+1},\;i=1,2,\cdots ,N-1,$$

where

$$\left\{ \begin{array}{c} {£}_{\varepsilon }^{N,M}{\hat{ℑ}}_{i}^{j+1}=\vartheta_{i}^{-}{\hat{ℑ}}_{i-1}^{j+1}+\vartheta_{i}^{c}{\hat{ℑ}}_{i}^{j+1}+\vartheta_{i}^{+}{\hat{ℑ}}_{i+1}^{j+1}\\ \vartheta_{i}^{-}=\frac{-\sigma \left(\rho \right)}{\rho }-\frac{3\omega_{1}\varpi_{i-1}^{j+1}}{2}-\omega_{2}\varpi_{i}^{j+1}+\frac{\omega_{1}\varpi_{i+1}^{j+1}}{2}+\ell \omega_{1}r_{i-1}^{j+1},\\ \vartheta_{i}^{c}=\frac{2\sigma \left(\rho \right)}{\rho }+2\omega_{1}\varpi_{i-1}^{j+1}-2\omega_{1}\varpi_{i+1}^{j+1}+2\ell \omega_{2}r_{i}^{j+1},\\ \vartheta_{i}^{+}=-\frac{-\sigma \left(\rho \right)}{\rho }-\frac{\omega_{1}\varpi_{i-1}^{j+1}}{2}+\omega_{2}\varpi_{i}^{j+1}+\frac{3\omega_{1}\varpi_{i-1}^{j+1}}{2}+\ell \omega_{1}r_{i+1}^{j+1},\\ H_{i}^{j+1}=\ell \left(\omega_{1}\varphi_{i-1}^{j+1}+\omega_{2}\varphi_{i}^{j+1}+\omega_{1}\varphi_{i+1}^{j+1}\right).\;\;\;\;\;\;\;\;\;\;\;\;\;\;\;\; \end{array}\right.$$

For sufficiently small mesh sizes the above matrix is non-singular and the matrix are diagonally dominant). Hence, by [26], the matrix ϑ is M-matrix and have an inverse. Therefore, the system of equations can be solved by matrix inverse with the given boundary conditions.

## Convergence analysis

Lemma 4.1 **Discrete Maximum Principle**

*Suppose that the discrete function* $\Psi_{i}^{j+1}$ *satisfies* $\Psi_{i}^{j+1}\geq 0$ *, on* ∂Υ *. Then* ${£}_{\varepsilon }^{N,M}\Psi_{i}^{j+1}\geq 0$ *on* $\Upsilon^{N,M}$ *implies that* $\Psi_{i}^{j+1}\geq 0$ *at each points of* ${\bar{\Upsilon }}^{N,M}.$

This lemma gives guarantee for the existence of unique discrete solution.

Lemma 4.2 **Discrete Uniform Stability**

*The solution*${\hat{ℑ}}_{i}^{j+1}$*of the discrete problem*(3.27)*at*(j+1)th*time level and*$\eta =\min_{0\leq i\leq N}\left\{r_{i}^{j+1}\right\}$*, where*η*is some positive constant satisfies*

$$∥{\hat{ℑ}}_{i}^{j+1}∥\leq \frac{∥{£}_{\varepsilon }^{N,M}{\hat{ℑ}}_{i}^{j+1}∥}{\eta }+\max\left\{\left|{\hat{ℑ}}_{0}^{j+1}\right|,\left|{\hat{ℑ}}_{N}^{j+1}\right|\right\}.$$

Proof Let define barrier functions ${\left(\Pi_{i}^{j+1}\right)}^{\pm }$ as

$${\left(\Pi_{i}^{j+1}\right)}^{\pm }=Z\pm {\hat{ℑ}}_{i}^{j+1},$$

where $Z=\frac{∥{£}_{\varepsilon }^{N,M}{\hat{ℑ}}_{i}^{j+1}∥}{\eta }+\max\left\{\left|{\hat{ℑ}}_{0}^{j+1}\right|,\left|{\hat{ℑ}}_{N}^{j+1}\right|\right\}$.

On the boundary points:

$${\left(\Pi_{0}^{j+1}\right)}^{\pm }=Z\pm {\hat{ℑ}}_{0}^{j+1}=\frac{∥{£}_{\varepsilon }^{N,M}{\hat{ℑ}}_{i}^{j+1}∥}{\eta }+\max\left\{\left|{\hat{ℑ}}_{0}^{j+1}\right|,\left|{\hat{ℑ}}_{N}^{j+1}\right|\right\}\pm \Theta^{j+1}\left(0\right)\geq 0,$$

$${\left(\Pi_{N}^{j+1}\right)}^{\pm }=Z\pm {\hat{ℑ}}_{N}^{j+1}=\frac{∥{£}_{\varepsilon }^{N,M}{\hat{ℑ}}_{i}^{j+1}∥}{\eta }+\max\left\{\left|{\hat{ℑ}}_{0}^{j+1}\right|,\left|{\hat{ℑ}}_{N}^{j+1}\right|\right\}\pm \Theta^{j+1}\left(N\right)\geq 0.$$

Now, on the discretized spatial domain $\Upsilon_{\ell }^{N}$:

$$\begin{array}{ccc} {£}_{\varepsilon }^{N,M}{\left(\Pi_{i}^{j+1}\right)}^{\pm } & = & {£}_{\varepsilon }^{N,M}\left(Z\pm {\hat{ℑ}}_{i}^{j+1}\right)\\ & = & \left(\frac{-\sigma \left(\rho \right)}{\rho }-\frac{3\omega_{1}\varpi_{i-1}^{j+1}}{2}-\omega_{2}\varpi_{i}^{j+1}+\frac{\omega_{1}\varpi_{i+1}^{j+1}}{2}+\ell \omega_{1}r_{i-1}^{j+1}\right)\left(Z\pm {\hat{ℑ}}_{i-1}^{j+1}\right)\\ & & +\;\left(\frac{2\sigma \left(\rho \right)}{\rho }+2\omega_{1}\varpi_{i-1}^{j+1}-2\omega_{1}\varpi_{i+1}^{j+1}+2\ell \omega_{2}r_{i}^{j+1}\right)\left(Z\pm {\hat{ℑ}}_{i}^{j+1}\right)\\ & & +\;\left(-\frac{-\sigma \left(\rho \right)}{\rho }-\frac{\omega_{1}\varpi_{i-1}^{j+1}}{2}+\omega_{2}\varpi_{i}^{j+1}+\frac{3\omega_{1}\varpi_{i-1}^{j+1}}{2}+\ell \omega_{1}r_{i+1}^{j+1}\right)\left(Z\pm {\hat{ℑ}}_{i+1}^{j+1}\right), \end{array}$$

$$\begin{array}{ccc} & = & \pm \left(\frac{-\sigma \left(\rho \right)}{\rho }-\frac{3\omega_{1}\varpi_{i-1}^{j+1}}{2}-\omega_{2}\varpi_{i}^{j+1}+\frac{\omega_{1}\varpi_{i+1}^{j+1}}{2}+\ell \omega_{1}r_{i-1}^{j+1}\right){ℑ}_{i-1}^{j+1}\\ & & \pm \left(\frac{2\sigma \left(\rho \right)}{\rho }+2\omega_{1}\varpi_{i-1}^{j+1}-2\omega_{1}\varpi_{i+1}^{j+1}+2\ell \omega_{2}r_{i}^{j+1}\right){\hat{ℑ}}_{i}^{j+1}\\ & & \pm \left(-\frac{-\sigma \left(\rho \right)}{\rho }-\frac{\omega_{1}\varpi_{i-1}^{j+1}}{2}+\omega_{2}\varpi_{i}^{j+1}+\frac{3\omega_{1}\varpi_{i-1}^{j+1}}{2}+\ell \omega_{1}r_{i+1}^{j+1}\right){ℑ}_{i+1}^{j+1}\\ & & +\;\left(\ell \omega_{1}r_{i-1}^{j+1}+2\ell \omega_{2}r_{i}^{j+1}+\ell \omega_{1}r_{i+1}^{j+1}\right)Z,\;\;\;\;\;\;\;\;\;\;\;\;\;\;\;\;\;\;\;\;\;\;\;\;\;\;\;\;\;\;\;\;\;\;\;\;\;\;\;\\ & & \pm \ell \left(\omega_{1}\varphi_{i-1}^{j+1}+2\omega_{2}\varphi_{i}^{j+1}+\omega_{1}\varphi_{i+1}^{j+1}\right)+\left(\ell \omega_{1}r_{i-1}^{j+1}+2\ell \omega_{2}r_{i}^{j+1}+\ell \omega_{1}r_{i+1}^{j+1}\right)Z,\;\;\;\;\\ & = & \left(\ell \omega_{1}r_{i-1}^{j+1}+2\ell \omega_{2}r_{i}^{j+1}+\ell \omega_{1}r_{i+1}^{j+1}\right)\left(\frac{∥{£}_{\varepsilon }^{N,M}{\hat{ℑ}}_{i}^{j+1}∥}{\eta }+\max\left\{\left|{\hat{ℑ}}_{0}^{j+1}\right|,\left|{\hat{ℑ}}_{N}^{j+1}\right|\right\}\right)\;\;\;\;\;\;\;\;\\ & & \mp \ell \left(\omega_{1}\varphi_{i-1}^{j+1}+2\omega_{2}\varphi_{i}^{j+1}+\omega_{1}\varphi_{i+1}^{j+1}\right),\;\;\;\;\;\;\;\;\;\;\;\;\;\;\;\;\;\;\;\;\;\;\;\;\;\;\;\;\;\;\;\;\;\;\;\;\;\;\;\;\;\;\;\;\;\;\;\;\;\;\;\;\;\;\;\;\;`\\ & & \geq 0,\;\;\;\;\;\;\;\text{since}\;\;r^{j+1}\left(z_{i}\right)\geq \eta >0.\;\;\;\;\;\;\;\;\;\;\;\;\;\;\;\;\;\;\;\;\;\;\;\;\;\;\;\;\;\;\;\;\;\;\;\;\;\;\;\;\;\;\;\;\;\;\;\;\;\;\;\;\;\;\;\;\;\;\;\; \end{array}$$

By Lemma 4.1, we obtain ${\left(\Pi_{i}^{j+1}\right)}^{\pm }\geq 0,0\leq i\leq 1$. Hence, the required bound is obtained. □

Lemma 4.3 *The local truncation error in space semi-discretization of the discrete problem*(3.27)*is given as*

$$\max_{i,j}\left|{\hat{ℑ}}^{j+1}\left(z_{i}\right)-{\hat{ℑ}}_{i}^{j+1}\right|\leq C\ell^{2},$$

*where*C*is a constant independent of*ε*and*ℓ*.*

Proof From the truncation error of Eq.  (3.19):

(4.1) $$\begin{array}{c} \left\{ \begin{array}{c} e_{i-1}^{\prime }=\frac{d{\hat{I}}^{j+1}\left(z_{i-1}\right)}{\mathrm{dz}}-\frac{d{\hat{I}}_{i-1}^{j+1}}{\mathrm{dz}}=\frac{\ell^{2}}{3}\frac{d^{3}{\hat{I}}^{j+1}\left(z_{i}\right)}{dz^{3}}-\frac{\ell^{3}}{12}\frac{d^{4}{\hat{I}}^{j+1}\left(z_{i}\right)}{dz^{4}}+\frac{\ell^{4}}{30}\frac{d^{5}{\hat{I}}^{j+1}\left(\xi_{i}\right)}{dz^{5}},\\ e_{i}^{\prime }=\frac{d{\hat{I}}^{j+1}\left(z_{i}\right)}{\mathrm{dz}}-\frac{d{\hat{I}}_{i}^{j+1}}{\mathrm{dz}}=-\frac{\ell^{2}}{6}\frac{d^{3}{\hat{I}}^{j+1}\left(z_{i}\right)}{dz^{3}}-\frac{\ell^{4}}{120}\frac{d^{5}{\hat{I}}^{j+1}\left(\xi_{i}\right)}{dz^{5}},\\ e_{i+1}^{\prime }=\frac{d{\hat{I}}^{j+1}\left(z_{i+1}\right)}{\mathrm{dz}}-\frac{d{\hat{I}}_{i+1}^{j+1}}{\mathrm{dz}}=\frac{\ell^{2}}{3}\frac{d^{3}{\hat{I}}^{j+1}\left(z_{i}\right)}{dz^{3}}+\frac{\ell^{3}}{12}\frac{d^{4}{\hat{I}}^{j+1}\left(z_{i}\right)}{dz^{4}}+\frac{\ell^{4}}{30}\frac{d^{5}{\hat{I}}^{j+1}\left(\xi_{i}\right)}{dz^{5}}, \end{array}\right. \end{array}$$

where $z_{i-1}<\xi <z_{i+1}$.

Substituting

$$\sigma \varepsilon \Xi_{\beta }=\varpi_{\beta }^{j+1}\frac{d{\hat{ℑ}}_{\beta }^{j+1}}{dz}+r_{\beta }^{j+1}{ℑ}_{\beta }^{j+1}-\varphi_{\beta }^{j+1},\;\;\beta =i,i\pm 1\;\;\;\;\;\;\;\;\;\;\;\;\;\;\;\;\;\;\;\;$$

into Eq.  (3.17) yields,

(4.2) $$\begin{array}{ccc} & & \sigma \varepsilon \left({\hat{ℑ}}_{i-1}^{j+1}-2{\hat{ℑ}}_{i}^{j+1}+{\hat{ℑ}}_{i+1}^{j+1}\right)=\ell^{2}\omega_{1}\left(\varpi_{i-1}^{j+1}\frac{d{\hat{ℑ}}_{i-1}^{j+1}}{dz}+r_{i-1}^{j+1}{\hat{ℑ}}_{i-1}^{j+1}-\varphi_{i-1}^{j+1}\right)\\ & & +\;2\ell^{2}\omega_{2}\left(\varpi_{i}^{j+1}\frac{d{\hat{ℑ}}_{i}^{j+1}}{dz}+r_{i}^{j+1}{\hat{ℑ}}_{i}^{j+1}-\varphi_{i}^{j+1}\right)+\ell^{2}\omega_{1}\left(\varpi_{i+1}^{j+1}\frac{d{\hat{ℑ}}_{i+1}^{j+1}}{dz}+r_{i+1}^{j+1}{\hat{ℑ}}_{i+1}^{j+1}-\varphi_{i+1}^{j+1}\right). \end{array}$$

Considering the corresponding exact solution to Eq. (4.2):

(4.3) $$\begin{array}{ccc} & & \sigma \varepsilon \left({\hat{ℑ}}^{j+1}\left(z_{i-1}\right)-2{\hat{ℑ}}^{j+1}\left(z_{i}\right)+{\hat{ℑ}}^{j+1}\left(z_{i+1}\right)\right)=\ell^{2}\omega_{1}\varpi^{j+1}\left(z_{i-1}\right)\frac{d{\hat{ℑ}}^{j+1}\left(z_{i-1}\right)}{dz}\\ & & +\;\ell^{2}\omega_{1}\left(r^{j+1}\left(z_{i-1}\right){\hat{ℑ}}^{j+1}\left(z_{i-1}\right)-\varphi^{j+1}\left(z_{i-1}\right)\right)+2\ell^{2}\omega_{2}\left(\varpi^{j+1}\left(z_{i}\right)\frac{d{\hat{ℑ}}^{j+1}\left(z_{i}\right)}{dz}+r^{j+1}\left(z_{i}\right){\hat{ℑ}}^{j+1}\left(z_{i}\right)\right)\\ & & -\;2\ell^{2}\omega_{2}g^{j+1}\left(z_{i}\right)+\ell^{2}\omega_{1}\left(\varpi^{j+1}\left(z_{i+1}\right)\frac{d{\hat{ℑ}}^{j+1}\left(z_{i+1}\right)}{dz}+r^{j+1}\left(z_{i+1}\right){\hat{ℑ}}^{j+1}\left(z_{i+1}\right)-\varphi^{j+1}\left(z_{i+1}\right)\right). \end{array}$$

Subtracting Eq.  (4.2) from Eq.  (4.3) and denoting $e_{\beta }={ℑ}^{j+1}\left(z_{\beta }\right)-{\hat{ℑ}}_{\beta }^{j+1}$,  for β=i,i±1 yields

(4.4) $$\begin{array}{ccc} & & \left(\sigma \varepsilon -\ell^{2}\omega_{1}r_{i-1}^{j+1}\right)e_{i-1}+\left(-2\sigma \varepsilon -2\ell^{2}\omega_{2}r_{i}^{j+1}\right)e_{i}+\left(\sigma \varepsilon -\ell^{2}\omega_{1}r_{i+1}^{j+1}\right)e_{i+1}\\ & & =\ell^{2}\left(\omega_{1}\varpi_{i-1}^{j+1}e_{i-1}^{\prime }+2\omega_{2}\varpi_{i}^{j+1}e_{i}^{\prime }+\omega_{1}\varpi_{i+1}^{j+1}e_{i+1}^{\prime }\right). \end{array}$$

Inserting Eq.  (4.1) in Eq.  (4.4) gives

(4.5) $$\begin{array}{ccc} & & \left(\sigma \varepsilon -\ell^{2}\omega_{1}r_{i-1}^{j+1}\right)e_{i-1}+\left(-2\sigma \varepsilon -2\ell^{2}\omega_{2}r_{i}^{j+1}\right)e_{i}+\left(\sigma \varepsilon -\ell^{2}\omega_{1}r_{i+1}^{j+1}\right)e_{i+1}\\ & & =\frac{\ell^{4}}{3}\left(\omega_{1}\varpi_{i-1}^{j+1}-\omega_{2}\varpi_{i}^{j+1}+\omega_{1}\varpi_{i+1}^{j+1}\right)\frac{d^{3}{ℑ}^{j+1}\left(z_{i}\right)}{dz^{3}}+\frac{\ell^{5}}{12}\left(-\omega_{1}\varpi_{i-1}^{j+1}+\omega_{1}\varpi_{i+1}^{j+1}\right)\frac{d^{4}{ℑ}^{j+1}\left(z_{i}\right)}{dz^{4}}\\ & & +\;\frac{\ell^{6}}{60}\left(2\omega_{1}\varpi_{i-1}^{j+1}-\omega_{2}\varpi_{i}^{j+1}+2\omega_{1}\varpi_{i+1}^{j+1}\right)\frac{d^{5}{ℑ}^{j+1}\left(\xi_{i}\right)}{dz^{5}}. \end{array}$$

Using the expressions $\varpi_{i-1}=\varpi_{i}-\ell \varpi_{i}^{{}^{\prime }}+\frac{\ell^{2}}{2!}\varpi^{\left(2\right)}\left(\xi_{i}\right)$ and $\varpi_{i+1}=\varpi_{i}+\ell \varpi_{i}^{\prime }+\frac{\ell^{2}}{2!}\varpi^{\left(2\right)}\left(\xi_{i}\right)$ in Eq. (4.5):

(4.6) $$\left(\sigma \varepsilon -\ell^{2}\omega_{1}r_{i-1}^{j+1}\right)e_{i-1}+\left(-2\sigma \varepsilon -2\ell^{2}\omega_{2}r_{i}^{j+1}\right)e_{i}+\left(\sigma \varepsilon -\ell^{2}\omega_{1}r_{i+1}^{j+1}\right)e_{i+1}=E_{i}\left(\ell \right),$$

where $E_{i}\left(\ell \right)=\frac{\ell^{4}}{3}\left(2\omega_{1}-\omega_{2}\right)\varpi_{i}^{j+1}\frac{d^{3}{ℑ}^{j+1}\left(z_{i}\right)}{dz^{3}}+O\left(\ell^{6}\right)$. Hence, for the choice of $\omega_{1}+\omega_{2}=1/2$, we obtain $E_{i}\left(\ell \right)=O\left(\ell^{4}\right)$.

The matrix representation of Eq. (4.6) is

(4.7) $$\left(\Gamma -\Omega \right)⊤=\hat{E},$$

where Γ=trid(−σε,2σε,−σε), $\Omega =trid\left(\ell^{2}\omega_{1}r_{i-1}^{j+1},2\ell^{2}\omega_{1}r_{i}^{j+1},\ell^{2}\omega_{1}r_{i+1}^{j+1}\right)$,

$⊤={\left[e_{1},e_{2},\cdots ,e_{N-1}\right]}^{t}$ and $\hat{E}={\left[-E_{1}\left(\ell \right),-E_{2}\left(\ell \right),\cdots ,-E_{N-1}\left(\ell \right)\right]}^{t}$.

Following [31], it can be shown that

(4.8) $$∥⊤∥\leq \frac{C}{\ell^{2}}\times O\left(\ell^{4}\right)=C\;\ell^{2},$$

where C is a constant, independent of ℓ and ε. □

Theorem 4.1 *Let*ℑ(z,t)*be the solution of problem*(3.4)*at each grid point*$\left(z_{i},t_{j+1}\right)$*and*${\hat{ℑ}}_{i}^{j+1}$*be its approximate solution obtained by the proposed scheme given in* Eq. (3.27)*. Then, the error estimate for the fully discrete method is given by*

$$\max_{i,j}\left|ℑ\left(z_{i},t_{j+1}\right)-{\hat{ℑ}}_{i}^{j+1}\right|\leq \left(C\right)\left(\tau +\ell^{2}\right).$$

Proof By combining the result of Lemmas 3.2 and 4.3 the required bound is obtained. □

## Numerical examples, results and discussion

Several model examples have been presented to illustrate the efficiency of the proposed method. As the exact solutions of the considered examples are not known, we calculate the maximum absolute error for each ε given in [4] by:

$$E_{\varepsilon }^{N,\tau }=\max_{\left(z_{i},t_{j+1}\right)\in \Upsilon^{N,M}}\left|{ℑ}^{N,\tau }\left(z_{i},t_{j+1}\right)-{ℑ}^{2N,\tau /2}\left(z_{i},t_{j+1}\right)\right|,$$

where $ℑ\left(z_{i},t_{j+1}\right)$ denotes the numerical solution obtained in $\Upsilon^{N,M}$ with N mesh intervals in the spatial direction and M mesh intervals in the temporal direction. We determine the corresponding order of convergence for each ε by:

$$R_{\varepsilon }^{N,\tau }=\log_{2}\left(E_{\varepsilon }^{N,\tau }/E_{\varepsilon }^{2N,\tau /2}\right).$$

Now, for each N and τ, the ε−uniform maximum point wise error is calculated using:

$$E^{N,\tau }=\max_{\varepsilon }E_{\varepsilon }^{N,\tau },$$

and the corresponding ε−uniform order of convergence is calculated by:

$$R^{N,\tau }=\log_{2}\left(E^{N,\tau }/E^{2N,\tau /2}\right).$$

Example 5.1 [23] Consider a singularly perturbed of the form in (1.1):

(5.1) $$\left\{ \begin{array}{c} \frac{\partial ℑ}{\partial t}-\varepsilon \frac{\partial^{2}ℑ}{\partial z^{2}}+ℑ\frac{\partial ℑ}{\partial z}-\left(1-ℑ\right)\left(ℑ-0.5\right)=0,\left(z,t\right)\in \Upsilon ,\\ ℑ\left(z,0\right)=z\left(1-z^{2}\right),0\leq z\leq 1,\\ ℑ\left(0,t\right)=0=ℑ\left(1,t\right)=0,t\in \chi_{t}. \end{array}\right.$$

Example 5.2 [23] Consider a singularly perturbed of the form in (1.1) :

(5.2) $$\left\{ \begin{array}{c} \frac{\partial ℑ}{\partial t}-\varepsilon \frac{\partial^{2}ℑ}{\partial z^{2}}+ℑ\frac{\partial ℑ}{\partial z}=0,\left(z,t\right)\in \Upsilon ,\\ ℑ\left(z,0\right)=z\left(1-z^{2}\right),0\leq z\leq 1,\\ ℑ\left(0,t\right)=0=ℑ\left(1,t\right)=0,t\in \chi_{t}. \end{array}\right.$$

Example 5.3 [14] Consider a singularly perturbed of the form in (1.1) :

(5.3) $$\left\{ \begin{array}{c} \frac{\partial ℑ}{\partial t}-\varepsilon \frac{\partial^{2}ℑ}{\partial z^{2}}+ℑ\frac{\partial ℑ}{\partial z}=\left(1-ℑ\right)\left(ℑ-0.5\right),\left(z,t\right)\in \Upsilon ,\\ ℑ(z,0)=\sin(\pi z),0\leq z\leq 1,\\ ℑ\left(0,t\right)=0=ℑ\left(1,t\right)=0,t\in \chi_{t}. \end{array}\right.$$

Example 5.4 [3] Consider a singularly perturbed of the form in (1.1) :

(5.4) $$\left\{ \begin{array}{c} \frac{\partial ℑ}{\partial t}-\varepsilon \frac{\partial^{2}ℑ}{\partial z^{2}}+ℑ\frac{\partial ℑ}{\partial z}=0,\left(z,t\right)\in \Upsilon ,\\ ℑ(z,0)=\sin(\pi z),0\leq z\leq 1,\\ ℑ\left(0,t\right)=0=ℑ\left(1,t\right)=0,t\in \chi_{t}. \end{array}\right.$$

**Fig. 1:**
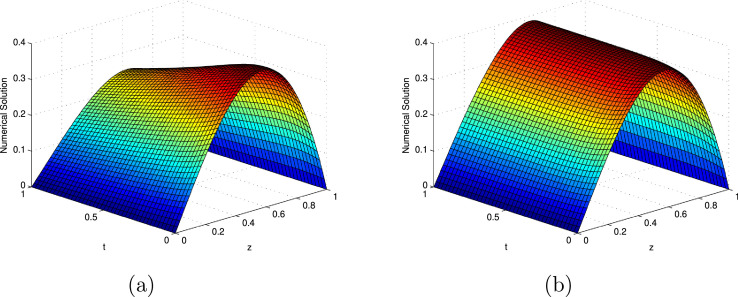
3D view of numerical solution for $N=64,M=40,\varepsilon =2^{-18}$: (a) Example 5.1, (b) Example 5.2.

**Fig. 2:**
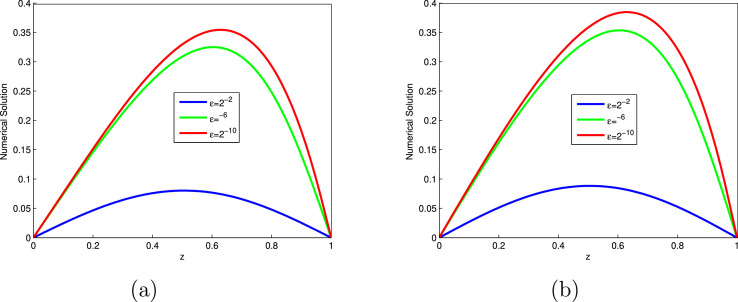
Effect of the ε on the behaviour of the solution with layer formation:(a) Example 5.1, (b) Example 5.2.

**Fig. 3:**
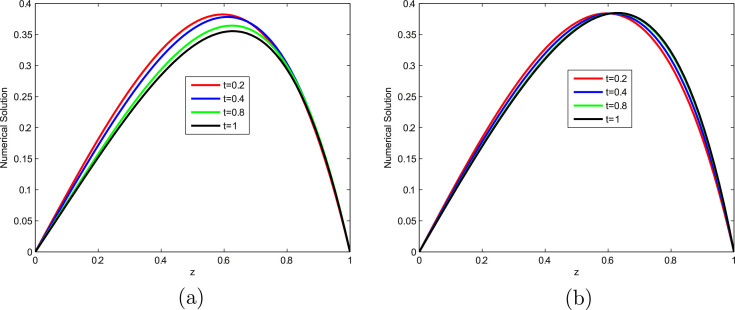
Numerical solution for $N=64,M=40,\varepsilon =2^{-16}$ and at different time levels: (a) Example 5.1, (b) Example 5.2.

**Fig. 4:**
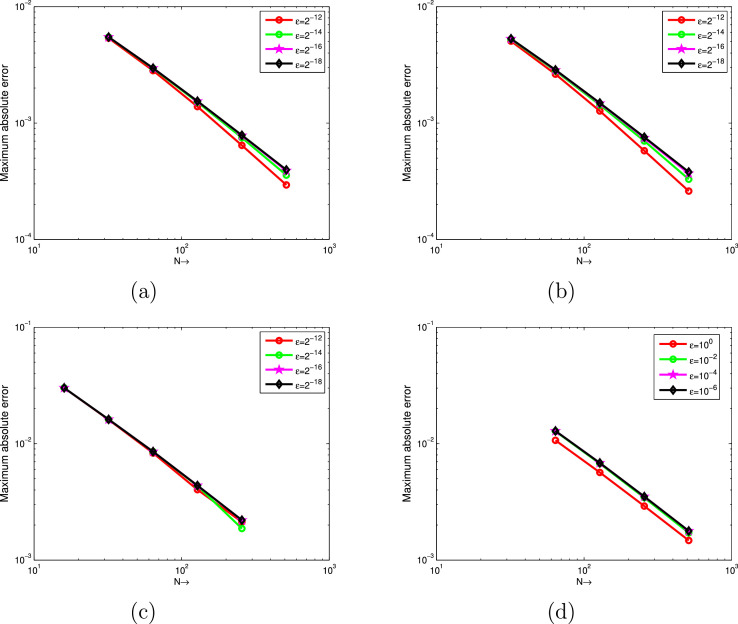
Example 5.1 on (a), Example 5.2 on (b), Example 5.2 on (c) and Example 5.2 on (d), Log-Log scale plot of the maximum absolute error for different values of ε.

The computed $E_{\varepsilon }^{N,\tau },E^{N,\tau }$ and $R^{N,\tau }$ of the considered problems using the proposed scheme are depicted in Table 1, Table 2, Table 3, Table 4, Table 5, Table 6, Table 7. From these tables, one can observes that the developed scheme converges independent of the perturbation parameter with order of convergence one. Concisely, the numerical results show that the proposed method provides better results than results in [14], [23]. In Fig. 1, the 3D view of the solution of Examples 5.1 and 5.2 are given for visualizing the boundary layer at N=64,M=40 and $\varepsilon =2^{-18}$. In Fig. 2, the effect of ε on the solution profile is shown. As one observes, as ε→0 strong boundary layer is created near z=1. In Fig. 3, the effect of the time step on the solution profile is shown. This figure shows the existence of the boundary layer at z=1 with time variable t→1. Fig. 4 shows the uniform convergence of the proposed scheme in log-log scale plot.

**Table 1:** Maximum absolute errors and rate of convergence for Example 5.1 with M=N.

ε↓N→	32	64	128	256	512
$2^{-6}$	1.8515e-03	9.3685e-04	4.6965e-04	2.3481e-04	1.1738e-04
$2^{-8}$	2.7447e-03	1.3157e-03	6.3201e-04	3.0830e-04	1.5206e-04
$2^{-10}$	3.6680e-03	1.7293e-03	7.7530e-04	3.5394e-04	1.6740e-04
$2^{-12}$	4.1483e-03	2.1406e-03	1.0250e-03	4.5790e-04	1.9969e-04
$2^{-14}$	4.2577e-03	2.2643e-03	1.1512e-03	5.6357e-04	2.6330e-04
$2^{-16}$	4.2648e-03	2.2811e-03	1.1781e-03	5.9538e-04	2.9515e-04
$2^{-18}$	4.2648e-03	2.2811e-03	1.1795e-03	5.9975e-04	3.0202e-04
$2^{-20}$	4.2648e-03	2.2811e-03	1.1795e-03	5.9975e-04	3.0241e-04
$E^{N,\tau }$	4.2648e-03	2.2811e-03	1.1795e-03	5.9975e-04	3.0241e-04
$R^{N,\tau }$	0.90275	0.95155	0.97574	0.98786

**Table 2:** Comparison of maximum absolute errors for Example 5.1 at the number of intervals N,M with results in [23].

ε	N=32	64	128	256	512	1024
↓	M=20	40	80	160	320	640
Present Method						
$2^{-6}$	2.8408e-03	1.4682e-03	7.4364e-04	3.7377e-04	1.8731e-04	9.3761e-05
$2^{-8}$	4.0156e-03	2.0020e-03	9.8557e-04	4.8685e-04	2.4170e-04	1.2040e-04
$2^{-10}$	4.8909e-03	2.4214e-03	1.1410e-03	5.4081e-04	2.6150e-04	1.2838e-04
$2^{-12}$	5.3650e-03	2.8201e-03	1.3852e-03	6.4340e-04	2.9416e-04	1.3782e-04
$2^{-14}$	5.4742e-03	2.9441e-03	1.5118e-03	7.4879e-04	3.5703e-04	1.6336e-04
$2^{-16}$	5.4827e-03	2.9641e-03	1.5399e-03	7.8120e-04	3.8923e-04	1.9002e-04
$2^{-18}$	5.4827e-03	2.9644e-03	1.5416e-03	7.8591e-04	3.9638e-04	1.9820e-04
Results in [23]						
$2^{-6}$	2.5289e-02	1.7672e-02	9.0066e-03	4.8378e-03	2.5035e-03	1.2731e-03
$2^{-8}$	3.8607e-02	1.9497e-02	1.1221e-02	6.2852e-03	3.3405e-03	1.7233e-03
$2^{-10}$	9.3183e-02	7.0120e-02	4.4773e-02	2.0546e-02	1.0545e-02	5.1608e-03
$2^{-12}$	1.7017e-01	1.0083e-01	6.2216e-02	3.9526e-02	2.0493e-02	1.0027e-02
$2^{-14}$	2.0410e-01	1.6703e-01	9.2110e-02	5.2580e-02	2.8766e-02	1.6523e-02
$2^{-16}$	2.0450e-01	1.5975e-01	1.2612e-01	6.9772e-02	3.8531e-02	2.0526e-02
$2^{-18}$	2.5614e-01	2.1031e-01	1.3406e-01	8.5618e-02	4.8834e-02	2.6219e-02

**Table 3:** Comparison of maximum absolute errors for Example 5.2 at the number of intervals N,M with results in [23].

ε	N=32	64	128	256	512	1024
↓	M=20	40	80	160	320	640
Present Method						
$2^{-6}$	2.2225e-03	1.1579e-03	5.8916e-04	2.9661e-04	1.4877e-04	7.4503e-05
$2^{-8}$	3.4104e-03	1.7015e-03	8.3475e-04	4.1158e-04	2.0411e-04	1.0162e-04
$2^{-10}$	4.4223e-03	2.1536e-03	1.0013e-03	4.7090e-04	2.2677e-04	1.1112e-04
$2^{-12}$	5.0534e-03	2.6284e-03	1.2705e-03	5.7857e-04	2.6065e-04	1.2122e-04
$2^{-14}$	5.2401e-03	2.8042e-03	1.4305e-03	7.0176e-04	3.2939e-04	1.4772e-04
$2^{-16}$	5.2884e-03	2.8530e-03	1.4768e-03	7.4538e-04	3.6902e-04	1.7849e-04
$2^{-18}$	5.3005e-03	2.8654e-03	1.4887e-03	7.5704e-04	3.8034e-04	1.8926e-04
Results in [23]						
$2^{-6}$	3.8767e-02	1.8983e-02	9.6122e-03	5.0867e-03	2.6211e-03	1.3306e-03
$2^{-8}$	4.4450e-02	2.0109e-02	1.0519e-02	5.9653e-02	3.1896e-03	1.6508e-03
$2^{-10}$	8.3339e-02	6.6120e-02	4.0769e-02	1.9284e-02	9.1775e-03	4.9998e-03
$2^{-12}$	1.8762e-01	8.4106e-02	5.7234e-02	3.309e-02	1.9041e-02	9.2260e-03
$2^{-14}$	1.9755e-01	1.5347e-01	8.3864e-02	4.6945e-02	2.5508e-02	1.4930e-02
$2^{-16}$	2.1340e-01	1.5016e-01	1.1582e-01	6.1958e-02	3.3441e-02	1.7672e-02
$2^{-18}$	2.8299e-01	1.7036e-01	1.1805e-01	7.4251e-02	4.1879e-02	2.2413e-02

**Table 4:** Comparison of rate of convergence for Example 5.1 at the number of intervals N,M with results in [23].

ε	N=32	64	128	256	512
↓	M=20	40	80	160	320
Present Method					
$2^{-6}$	0.9522	0.9814	0.9925	0.9967	0.99837
$2^{-8}$	1.0042	1.0224	1.0175	1.0103	1.0054
$2^{-10}$	1.0143	1.0855	1.0771	1.0483	1.0264
$2^{-12}$	0.9278	1.0257	1.1063	1.1291	1.0938
$2^{-14}$	0.8948	0.9616	1.0136	1.0685	1.1280
$2^{-16}$	0.8873	0.9448	0.9791	1.0051	1.0345
$2^{-18}$	0.8871	0.9433	0.9720	0.9875	0.9999
Results in [23]					
$2^{-6}$	0.5170	0.9724	0.8966	0.9504	0.9756
$2^{-8}$	0.9856	0.7971	0.8362	0.9119	0.9549
$2^{-10}$	0.4102	0.6472	1.1238	0.9623	1.0309
$2^{-12}$	0.7550	0.6966	0.6544	0.9477	1.0312
$2^{-14}$	0.2892	0.8587	0.8088	0.8701	0.7999
$2^{-16}$	0.3563	0.3410	0.8540	0.8566	0.9086
$2^{-18}$	0.2844	0.6496	0.6469	0.8100	0.8973

**Table 5:** Comparison of rate of convergence for Example 5.2 at the number of intervals N,M with results in [23].

ε	N=32	64	128	256	512
↓	M=20	40	80	160	320
Present Method					
$2^{-6}$	0.9407	0.9748	0.9901	0.9955	0.9977
$2^{-8}$	1.0031	1.0274	1.0202	1.0118	1.0062
$2^{-10}$	1.0380	1.1049	1.0884	1.0542	1.0291
$2^{-12}$	0.9431	1.0488	1.1348	1.1504	1.1045
$2^{-14}$	0.9020	0.9711	1.0275	1.0912	1.1569
$2^{-16}$	0.8904	0.9500	0.9864	1.0143	1.0479
$2^{-18}$	0.8874	0.9447	0.9756	0.9931	1.0069
Results in [23]					
$2^{-6}$	1.0301	0.9818	0.9181	0.9566	0.9781
$2^{-8}$	1.1443	0.9348	0.8183	0.9032	0.9502
$2^{-10}$	0.3339	0.6976	1.0801	1.0712	0.8762
$2^{-12}$	1.1575	0.5553	0.5792	1.0086	1.0453
$2^{-14}$	0.3643	0.8718	0.8371	0.8800	0.7727
$2^{-16}$	0.5076	0.3746	0.9025	0.8897	0.9202
$2^{-18}$	0.7322	0.5292	0.6689	0.8262	0.9019

**Table 6:** Comparison of maximum absolute errors for Example 5.3 at τ=0.001 with results in [14].

ε↓N→	16	32	64	128	256
$2^{-6}$	1.1441e-02	3.3769e-03	1.0928e-03	5.2467e-04	2.5971e-04
$2^{-8}$	2.4264e-02	1.0286e-02	3.5936e-03	1.2628e-03	5.5544e-04
$2^{-10}$	2.9305e-02	1.4883e-02	7.0408e-03	3.0236e-03	1.2559e-03
$2^{-12}$	3.0111e-02	1.6059e-02	8.3119e-03	4.0215e-03	2.1264e-03
$2^{-14}$	3.0130e-02	1.6165e-02	8.5381e-03	4.3332e-03	1.8652e-03
$2^{-16}$	3.0130e-02	1.6165e-02	8.5447e-03	4.3660e-03	2.2016e-03
$2^{-18}$	3.0130e-02	1.6165e-02	8.5447e-03	4.3661e-03	2.2042e-03
$2^{-20}$	3.0130e-02	1.6165e-02	8.5447e-03	4.3661e-03	2.2042e-03
$E^{N,\tau }$	3.0130e-02	1.6165e-02	8.5447e-03	4.3661e-03	2.2042e-03
$R^{N,\tau }$	0.89833	0.91977	0.96868	0.98609	
$E^{N,\tau }$[14]					
	4.1129e-2	2.2723e-2	1.2008e-2	6.179e-3	3.136e-3

**Table 7:** Comparison of maximum absolute errors for Example 5.4 at the number of intervals N,M with results in [3].

ε	N=64	128	256	512
↓	M=20	40	80	160
Present Method				
$10^{0}$	1.0681e-02	5.6600e-03	2.9086e-03	1.4738e-03
$10^{-2}$	1.2760e-02	6.7431e-03	3.4351e-03	1.7144e-03
$10^{-4}$	1.2850e-02	6.8269e-03	3.5117e-03	1.7800e-03
$10^{-6}$	1.2850e-02	6.8276e-03	3.5125e-03	1.7808e-03
Results in [3]				
$10^{0}$	1.070e-02	2.336e-03	5.212e-04	1.290e-04
$10^{-2}$	2.550e-02	5.796e-03	1.409e-03	3.555e04
$10^{-4}$	9.929e-02	7.841e-02	6.262e-02	4.828e-02
$10^{-6}$	9.929e-02	7.841e-02	6.260e-02	4.979e-02

## Conclusion

In this paper, uniformly convergent computational scheme is developed, analyzed, and implemented for solving singularly perturbed non-linear Burgers’ problem which has a boundary layer. The developed scheme constitutes the implicit Euler in the time discretization and the fitted exponential cubic spline method in space discretization. It is shown that the developed scheme converges ε−uniformly which is first-order accurate in time and second-order accurate in space. Four test examples are considered to validate the theoretical findings. The results in the tables and figures indicate the parameter uniform convergence of the scheme with the rate of convergence of one. Also, the experiment shows that the proposed scheme provides better numerical accuracy compared to schemes in [3], [14], [23]. From the results presented, one can conclude that the proposed scheme is an efficient robust numerical scheme to approximate the solution of the singularly perturbed unsteady non-linear Burgers’ equation. The scheme can also be extendable for solving higher order families of singularly perturbed partial differential problems and singularly perturbed problems with degenerate coefficients. The obtained numerical results and graphs are plotted by using the MATLABR2013a software package.

## Funding statement

This research did not receive any specific grant from funding agencies.

## Data availability

Uniformly Convergent Computational Method for Singularly Perturbed Unsteady Burger-Huxley Equation

## CRediT authorship contribution statement

**Imiru Takele Daba:** Investigation, Formal analysis, Software, Writing – original draft, Writing – review & editing. **Gemechis File Duressa:** Investigation, Formal analysis, Software, Writing – original draft.

## Declaration of Competing Interest

The authors declare that they have no conflicts of interest.
